# Correlates of a single cortical action potential in the epidural EEG

**DOI:** 10.1016/j.neuroimage.2014.12.057

**Published:** 2015-04-01

**Authors:** Bartosz Teleńczuk, Stuart N Baker, Richard Kempter, Gabriel Curio

**Affiliations:** a Unité de Neurosciences, Information et Complexité, Centre National de la Recherche Scientifique, 91198 Gif-sur-Yvette, France; b Neurophysics Group, Department of Neurology, Campus Benjamin Franklin, Charité-Universitätsmedizin Berlin, 12200 Berlin, Germany; c Department of Biology, Institute for Theoretical Biology, Humboldt-Universität zu Berlin, 10115 Berlin, Germany; d Institute of Neuroscience, Newcastle University, Newcastle upon Tyne NE2 4HH, UK; e Bernstein Center for Computational Neuroscience Berlin, 10115 Berlin, Germany; f Bernstein Focus Neurotechnology Berlin, 10587 Berlin, Germany

**Keywords:** Single-unit activity, EEG, Spike-triggered average, Somatosensory cortex

## Abstract

To identify the correlates of a single cortical action potential in surface EEG, we recorded simultaneously epidural EEG and single-unit activity in the primary somatosensory cortex of awake macaque monkeys. By averaging over EEG segments coincident with more than hundred thousand single spikes, we found short-lived (≈ 0.5 ms) triphasic EEG deflections dominated by high-frequency components > 800 Hz. The peak-to-peak amplitude of the grand-averaged spike correlate was 80 nV, which matched theoretical predictions, while single-neuron amplitudes ranged from 12 to 966 nV. Combining these estimates with post-stimulus-time histograms of single-unit responses to median-nerve stimulation allowed us to predict the shape of the evoked epidural EEG response and to estimate the number of contributing neurons. These findings establish spiking activity of cortical neurons as a primary building block of high-frequency epidural EEG, which thus can serve as a quantitative macroscopic marker of neuronal spikes.

## Highlights

• Cortical spikes are coincident with short-lived (~ 0.5 ms) EEG deflections.
• Cortical spikes produce ~  80 nV epidural EEG deflections at a distance of ~ 5 mm.
• EEG potentials due to spikes are dominated by high-frequency (> 800 Hz) components.
• High-frequency (> 800 Hz) EEG is a genuine macroscopic marker of spiking activity.

## Introduction

Electric surface potentials recorded from the scalp (electroencephalography, EEG; Berger, 1929), from the pia mater (electrocorticography; Foerster and Altenburger, 1935), or from the dura mater (epidural EEG; Noachtar and Rémi, 2009) provide access to neuronal activity in humans and animal models. These macroscopic signals have proven indispensable in clinical diagnostics, in research on neuronal correlates of cognitive processes (Friston, 2009) and for operating brain–computer interfaces (Birbaumer et al., 2008). Such widespread applications are possible owing to the high temporal resolution of scalp potentials, which enable access to signal frequencies as high as 600 Hz (Curio et al., 1994; Hashimoto et al., 1996) or even at and above 1000 Hz (Fedele et al., 2012).

The cellular mechanisms generating macroscopic surface potentials > 400 Hz are not yet fully understood; in particular, spikes are commonly thought not to contribute substantially to the scalp EEG (Nunez and Srinivasan, 2005; Niedermeyer and Silva, 2004). EEG activity at frequencies below 100 Hz reflects slowly-changing processes (10–100 ms), such as post-synaptic potentials or spike after-hyperpolarizations (Storm, 1987). The magnitude and sign of these contributions depend on cell morphology (Lindén et al., 2010), cortical anatomy (Klee and Rall, 1977), localization of synaptic inputs along the somato-dendritic axis (Creutzfeldt et al., 1966), and synchrony between active neurons (Kuokkanen et al., 2010; Lindén et al., 2011; Ray et al., 2008). These factors hinder the interpretation of low-frequency surface potentials in terms of microscopic neuronal processes: For example, excitatory and inhibitory post-synaptic potentials located, respectively, at the apical dendrites and at the soma may be associated with identical surface depolarizations (da Silva and van Rotterdam, 2004). Notably, if EEG activity > 400 Hz reflects spiking activity, it might inform about the output of the excitatory/inhibitory balance (Ray et al., 2013).

To make this feasible, high-fidelity EEG recording systems have been developed allowing low-noise recordings also at frequencies above 1000 Hz (Fedele et al., 2012; Scheer et al., 2009) thus covering the spectrum of extracellular action potentials (Fee et al., 1996; Belluscio et al., 2012). High-fidelity EEG, therefore, provides a unique opportunity to observe non-invasively the output of neuronal computation–spikes. Recently, we found that stimulus-evoked EEG bursts > 400 Hz correlated with spikes of single cortical neurons (Baker et al., 2003) and its amplitude co-varied with the temporal spike patterns on a single-trial basis (Telenczuk et al., 2011). Spike-related activity was also reported in lower frequency bands, such as gamma and high-gamma (Whittingstall and Logothetis, 2009; Manning et al., 2009; Waldert et al., 2013). Modelling studies suggest that high-frequency EEG components might be generated by synchronous spikes of pyramidal neurons (Murakami and Okada, 2006; Ray et al., 2008).

To interpret these findings in terms of population spike rate requires a quantitative link between the amplitude of surface potentials and the underlying single-cell spike activation. Specifically, the question “What is the contribution of a *single* cortical action potential to epidural EEG signals?” awaits an empirical answer. Here, we address this question by combining extensive simultaneous recordings of epidural EEG and single-unit activity in macaque somatosensory cortex. Our experimental estimate of single-spike correlates in the epidural EEG matches theoretical predictions and allows for extrapolation to evoked surface potentials in non-human primates and humans.

A preliminary report of these results has been published in abstract form in Telenczuk et al. (2012).

## Methods

### Experimental protocol

We re-analyzed data from Baker et al. (2003) where details of the experimental protocol can be found. Briefly, neuronal responses were evoked in the hand representation of the primary somatosensory cortex of two awake Macaca mulatta monkeys by electrical median-nerve stimulation at the wrist (pulse width: 0.2 ms; repetition rate: 3 Hz; intensity: 150% motor threshold). The monkeys continued to perform a behavioural task (precision grip with the non-stimulated hand; Baker et al., 2003) during the stimulation.

Extracellular potentials were recorded using a 16-channel ‘Eckhorn’ drive (Thomas Recording GmbH, Giessen, Germany). The skull at the recording site was trepanated, the dura thinned and treated every day with anti-mitotic agent to allow penetration by the electrodes and prevent regrowth. Each of the platinum/glass electrodes (electrode impedance: 1 MΩ) was shielded against receiving/emitting interferences by metal guide tubes. To record extracellular action potentials, the electrodes were advanced individually into the cortex (Brodmann area 3b) until a well-isolated single-unit responsive to median nerve stimulation was found. Each recording site was used for about a week after which the state of the cortex deteriorated and a new site was prepared. In total, 6 and 4 penetration sites were used in each animal subject, respectively. Extracellular potentials referenced to the headstage were first band-pass filtered (1–10 kHz) and then continuously sampled at 24 kHz for a period of approximately 1000 stimulus repetitions (median: 1031 trials, range: 150–2128 trials).

Receptive fields of recorded cells were tested by means of manual tapping using a stylus. All cells responded to touch of the radial part of the palm or palmar surface of the thumb, index or middle finger, i.e., in the skin territory innervated by the median nerve.

EEG was measured at the surface of the dura with two ball electrodes made of silver wire with a tip diameter of about 2 mm and separated by about 10 mm. The recording chamber was fixed to the bone by means of acrylic. To avoid short-circuit between and around electrodes the chamber was made of plastic and filled with oil for the duration of the recording to prevent short-circuit and fluid accumulation. The signals were recorded in a bipolar montage using a two-stage amplification that was separated from the micro-electrodes recordings. Epidural EEG signals were band-pass filtered (3–3000 Hz) and sampled at 6 kHz. The precise position of the electrodes varied from session to session, but they always spanned the posterior and anterior edges of the central sulcus. The polarity of the averaged somatosensory evoked potential (SEP) defined at the latency of 10 ms was always up reflecting positivity (negativity) at the pre-central (post-central) electrode of the bipolar montage (see the first SEP peak in Fig. 3b). Consistently with the awake state of the subjects, epidural EEG signals were desynchronized during the entire period of the recording, i.e. it displayed a much higher power of fast oscillations (15–75 Hz) compared to slow oscillations (1–10 Hz) after subtracting the evoked response.

The stimulus-evoked burst of high-frequency EEG activity (Fig. 5c, thin line) was extracted by averaging band-pass filtered (800–3000 Hz) responses in the time window 0–20 ms post-stimulus over all stimulation trials and all recording sessions.

Digital filtering was performed off-line with a non-causal phase-preserving filter in the Fourier domain. To avoid ringing artefacts (Widmann and Schröger, 2012) we smoothed the transition between pass- and stop-bands with a Gaussian (half-width 117 Hz).

### Spike discrimination

Action potentials were detected by amplitude thresholding of extracellular potentials; the threshold was chosen manually to detect only spikes substantially above the noise level (about 2 s.d. of background noise). All detected spikes were aligned to the first negative peak of the spike waveform. The waveforms were parameterized by their peak-to-peak amplitude, width at defined fraction of full amplitude, and projection coefficients on two main principal components. The spike timings of single units were discriminated based on these shape features using a manual cluster-cutting method that allowed for identification of clusters of arbitrary shapes. To ensure correct clustering, the procedure was performed independently by two operators using two different software packages (GetSpike, and SpikeSort available at http://spikesort.org), and potential inconsistencies were resolved by identifying the spike waveform visually in the raw microelectrode recordings.

To validate the spike discrimination, we checked the extracellular action potentials generated by a putative single cell for consistency of its waveform and amplitude over the full recording period of that cell. Spike trains with evidence of poor spike sorting (inconsistent waveforms or interspike intervals < 1 ms) were excluded from subsequent analyses.

The depth of discriminated cells was given by the distance travelled by the tip of the electrode from the dura.

### Spike-triggered EEG average

To identify single-spike contributions to the epidural EEG, we computed the average EEG around a spike, which is called the “spike-triggered average”. To avoid overlap of contributions from multiple action potentials, we removed spikes that were separated by less than 6 ms (for high-frequency EEG) or 30 ms (for wideband EEG). Next, we extracted segments of on-going epidural EEG centred on each of the spikes (± 3 ms or ± 15 ms around each selected spike's first negative peak, respectively). To allow for a more precise alignment of spikes and the EEG, the on-going EEG was upsampled to 10 kHz using linear interpolation. The average of all extracted segments is the spike-triggered EEG average (EEG-STA), which estimates the single-spike correlate in the epidural EEG.

**Fig. 1:**
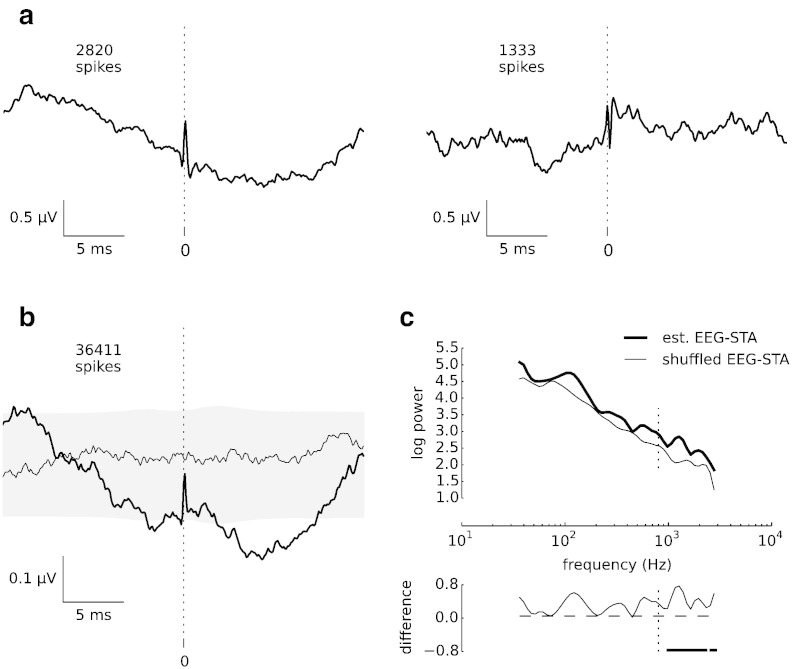
Spike-triggered average of epidural EEG (EEG-STA). (a) EEG-STAs of wideband EEG (3–3000 Hz) for two sample cells. A sharp transient of epidural EEG activity that coincides with cellular action potentials centred at time lag 0 (dotted lines) rides on top of slower (10–30 ms) fluctuations. The numbers of spikes used to calculate the EEG-STAs are given in the top-left corner. (b) Grand average EEG-STA (bold line) pooled across 40 neurons identified in two monkeys (monkey A: 29 neurons, 26 477 spikes, median 627 spikes/neuron, range 76–2888 spikes/neuron; monkey B: 11 neurons, 9934 spikes, median 709 spikes/neuron, range 189–2915 spikes/neuron). Spike-correlated EEG components are abolished when spikes are randomly shuffled across trials (thin line, shuffled EEG-STA). Confidence intervals for shuffled EEG-STA are shown in grey shading (confidence level 99%). (c) Comparison of EEG-STA spectra before (top: bold line) and after (top: thin line) shuffling spikes across trials. The spike-related component in the unshuffled EEG-STA causes an increase in signal power (bottom: difference of log-spectra), which reaches significance level (horizontal bars; p < 0.01, bootstrap test) in the high-frequency band (> 800 Hz, delimited by dotted line).

Evoked responses, even in the absence of spikes, introduce potential shifts (baseline potential) that might contaminate the EEG-STA. To correct the baseline, we recalculated the EEG-STA after shuffling spikes across trials (shift predictor) (Schwartz et al., 2006). Next, we subtracted the mean of such 100 shuffled EEG-STAs from the estimated EEG-STA. Baseline correction was not applied to the EEG-STA calculated in the late window (200–290 ms, Fig. 3e, upper panel) and in the spectral analysis of wideband EEG-STA (Fig. 1c).

To calculate the high-frequency EEG-STA, prior to averaging we filtered epidural EEG within the band 800–3000 Hz using the non-causal phase-preserving filter described above.

### Statistical methods

To test the significance of observed EEG-STA peaks, we recalculated the EEG-STA after shuffling spikes across trials. From these shuffled EEG-STAs we calculated confidence intervals for each lag separately; the upper and lower limits of a confidence interval were calculated as ± 2.58 times the standard error of the mean across spike-triggered segments (99% confidence limits).

The significance of peaks of the single-cell high-frequency EEG-STA was tested by means of a two-sample bootstrap test. First, we compared the maximum absolute amplitude of single-cell EEG-STA within the window from − 0.3 to 0.5 ms (8 samples at 10 kHz) prior to and after shuffling spikes across trials. If the peak amplitudes before and after shuffling were not significantly different, the peak might have just been a random fluctuation. To assess its statistical significance, we estimated the empirical distribution of (shuffled and unshuffled) peak amplitudes from 5000 different EEG-STAs formed from random combinations of spike-triggered epidural EEG segments. The estimated p-value was taken as the fraction of EEG-STA instances in which the amplitude after shuffling spikes across trials was greater than before shuffling. No correction for multiple comparisons was performed.

To test for potential spike-rate trends that were present in the late period (200–290 ms post-stimulus), we pooled spikes elicited across all trials into a single spike train, and the resulting spike times were compared against a uniform distribution using a quantile plot and the Kolmogorov–Smirnoff test.

### Spectral analysis

**Fig. 2:**
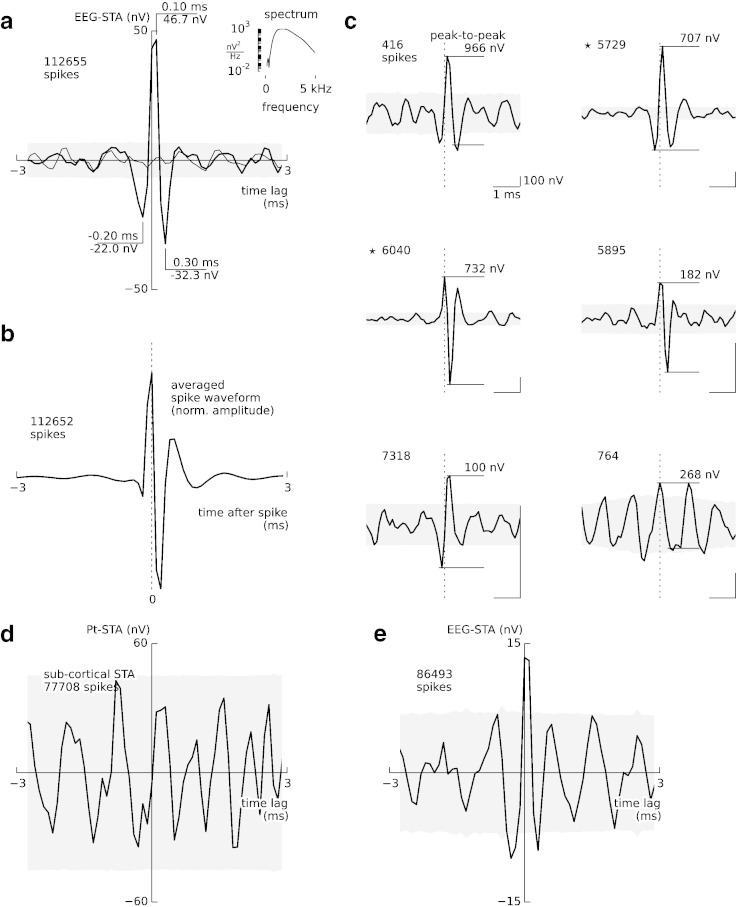
Correlate of a single action potential in high-frequency (> 800 Hz) epidural EEG. (a) The grand average EEG-STA of high-pass filtered epidural EEG (EEG burst, 800–3000 Hz) is triphasic and has a short (< 1 ms) duration (peak latencies and amplitudes annotated in the plot). Randomly shuffling spikes across trials abolishes the EEG-STA peaks (thin line). The grey-shaded area indicates the 99% confidence interval for uncorrelated spikes/EEG (see the Methods section). The minimum interspike interval (ISI > 6 ms) was adjusted to the smaller EEG-STA window size resulting in a larger number of averaged segments. Inset: power spectrum of the estimated EEG-STA. (b) Grand average of high-pass filtered (> 800 Hz) extracellular spike waveforms (negative peaks point upwards). To compensate for possible amplitude differences related to the position of the electrode relative to the neuron, spike waveforms were normalised at the negative peak to 1. This normalisation was used only here for the purpose of visualisation, and not in any other analyses. (c) EEG-STAs of those six cells showing at least one significant peak at latencies between − 0.3 ms and 0.5 ms (two-sample bootstrap, p < 0.01) sorted according to decreasing p-values (left-to-right, top-to-bottom). Grey-shaded area: 99% confidence interval. Stars mark sample cells used for calculation of wideband EEG-STA in Fig. 1a. (d) The STA derived from a field electrode placed in the brainstem (pyramidal tract, Pt-STA) that shared the acquisition pathway with epidural electrodes. The Pt-STA did not show a significant spike-related potential (grand average, solid line, with confidence intervals, grey area). (e) Grand-average EEG-STA for the remaining cells (not included in b, 34 out of 40 cells).

To calculate the spectra of the EEG-STAs (Fig. 1c and inset of Fig. 2a) we selected frequencies (ranging from 0 to Nyquist frequency) equidistant on a logarithmic scale (8 frequencies per octave). To minimize spectral leakage, for each frequency *f_i_* we truncated the EEG-STA so that its length was an integer multiple of the period 1/*f_i_* and then we multiplied the data points by a Hanning window function of the same length. The Fourier coefficients were calculated by direct projection on a sine and cosine of frequency *f_i_*. Finally, we smoothed the spectrum using a moving average in the frequency domain (the length of the smoothing window was half an octave).

To test the significance of the differences between shuffled and unshuffled spectra (Fig. 1), we used a bootstrapping technique. First, we calculated 500 spectra from EEG-STAs based on random samples (drawn with repetition) of the EEG segments triggered on shuffled spikes (see the previous section). For each frequency a two-tailed p-value was obtained from the fraction of such “shuffled spectra” that were higher/lower than the unshuffled spectrum.

The spectra of the post-stimulus time histogram (Fig. 5b) and the predicted and modelled high-frequency EEG burst (Fig. 5d) was estimated using a multi-taper method (bandwidth 600 Hz).

### Contribution of correlated cell activity

We calculated the contribution of possible neuronal correlations to the spike-triggered EEG average based on the present estimated EEG-STA and published data on neuronal cross-correlations in the cortex. First, previously published experimental cross-correlograms were approximated with a Gaussian function:

(1) $$f_{xcf}\left(t\right)=a_{xcf}e^{-t^{2}/b_{xcf}}\text{.}$$

The parameters *a*_xcf_ and *b*_xcf_ were selected such that the peak correlation, *A*_xcf_ = *a*_xcf_, and half-amplitude width, $T_{xcf}=\sqrt{2b_{xcf}\ln2}$, agreed with experimental data (Jermakowicz et al., 2009; *A*_xcf_ = 0.008 coincidences/s and *T*_xcf_ = 17 ms).

Next, we approximated the contribution of a single neuron to the epidural EEG with a Mexican hat function:

(2) $$g_{STA}\left(t\right)=\frac{a_{STA}}{b_{STA}}\left(2-4\frac{{\left(t-t_{STA}\right)}^{2}}{b_{STA}}\right)e^{-\frac{{\left(t-t_{STA}\right)}^{2}}{b_{STA}}}\text{.}$$

The function consists of one upward-going peak with the latency *t*_STA_ and amplitude *A*_STA_ = 2*a*_STA_/*b*_STA_, and two downward-going side lobes separated by $T_{STA}=\sqrt{6b_{STA}}$ (see Fig. 4a, bold line). To find the parameters *a*_STA_, *b*_STA_ and the peak latency *t*_STA_, the function was fitted to the grand-average high-frequency EEG-STA (Figs. 2a and 4a, grey line).

Finally, we calculated the predicted EEG contribution from spikes correlated with the spikes of the “trigger” neuron (“correlation” EEG-STA, cSTA):

(3) $$g_{\mathrm{cSTA}}\left(t\right)=Nf_{xcf}\left(t\right)*g_{STA}\left(t\right)\text{,}$$

where *N* is the number of contributing neurons and ∗ denotes the convolution. It can be shown that *g*_cSTA_(*t*) is also a Mexican hat function with amplitude $A_{\mathrm{cSTA}}=2\sqrt{\pi }Na_{xcf}a_{STA}\sqrt{\frac{b_{xcf}b_{STA}}{{\left(b_{xcf}+b_{STA}\right)}^{3}}}$ and width $T_{\mathrm{cSTA}}=\sqrt{6\left(b_{xcf}+b_{STA}\right)}$. These results quantify the effect of the potential correlations between multiple neurons on the EEG-STA estimate.

### Prediction of high-frequency EEG waveforms

To predict somatosensory-evoked EEG bursts from neuronal spikes, we convolved single-neuron post-stimulus time histograms (PSTHs, bin width 0.1 ms) with the respective single-neuron EEG-STAs. In most of the sessions, we recorded the activity of only a single neuron from the responding population; to approximate the mean response of the whole population, we averaged EEG contributions of neurons recorded across all the sessions.

The statistical error in the predicted EEG burst may originate from noise in estimation of EEG-STA and from limited sampling of neurons used to estimate the population response. To derive confidence intervals that take into account both sources of variability, we used the bootstrap method. This method allows the estimation of the empirical distribution of EEG burst amplitudes based on a repeated sampling from the available dataset. In the first step of the analysis, we randomly selected a single cell from the dataset. Next, we calculated the EEG-STA from spikes drawn randomly with repetition from all spikes of this cell, such that the total number of averaged segments was conserved, and we convolved it with the cell's PSTH. We repeated the procedure as many times as the total number of cells (by drawing 40 cells with repetition) and then averaged the results, which gave us a single estimate of an EEG burst. To obtain the amplitude distribution, we calculated 500 of such EEG-burst estimates. Time-dependent confidence intervals were then obtained by taking 5% and 95% percentiles of this distribution at each time point separately.

## Results

### Epidural EEG potentials are correlated with spikes of cortical neurons

To determine the correlate of a single spike in the epidural EEG, we recorded the local epidural EEG and single-unit activity from neurons localised in the somatosensory cortex (area 3b) of two *Macaca mulatta* monkeys (29 neurons from monkey A, 11 neurons from monkey B). We determined the timing of extracellular action potentials of single neurons by means of an off-line spike sorting procedure (see the Methods section), and we extracted non-overlapping segments (30 ms long) of simultaneously measured epidural EEG signals centred on each spike. The average of such segments represents an EEG spike-triggered average (EEG-STA), i.e., the mean contribution of a single action potential to the macroscopic EEG activity as a function of time before (negative time lag) or after the spike (positive time lag). Such a contribution is expected to be exceedingly small compared to the on-going EEG, which sums contributions of a large number of neurons and non-neural activity, such as muscle activity, environmental noise, band-limited amplifier noise, etc. Nevertheless, we observed distinct EEG fluctuations accompanying single action potentials; two examples are shown in Fig. 1a. In particular, on top of slow fluctuations (with a period of around 10–30 ms), we found a sharp transient of duration below 1 ms that was coincident with the cellular action potential.

The features observed in the single-cell examples were preserved in the grand average of spike-triggered EEG segments pooled from all single units in both monkeys (40 cells; Fig. 1b, bold line). The sharp transient was abolished after shuffling spikes randomly across trials (shift predictor, Fig. 1b, thin line; grey-shaded area: 99% confidence interval).

### Spikes contribute to fast EEG potentials (above 800 Hz)

A spectral analysis of the grand-average EEG-STA (Fig. 1c) exposed the sharp transient as a significant excess of signal power at frequencies > 800 Hz (thick line) as compared to the spectrum of the shift predictor (thin line). Since the power spectra of the epidural EEG potentials derived from synaptic potentials and sub-threshold activity are known to be dominated by frequencies below 200 Hz (Mitzdorf, 1985), we conjecture that such high-frequency components particularly reflect neuronal spikes (Baker et al., 2003). In further analyses we therefore focused on high-frequency EEG.

To better isolate the contribution of action potentials to epidural EEG, we repeated the spike-triggered analysis for high-pass filtered EEG (800–3000 Hz) and decreased the size of the EEG-STA window to focus on the sharp transient identified above. This decrease of the window size from 30 ms to 6 ms increased considerably the number of non-overlapping EEG segments that entered the analysis (Table 1). The resulting grand-average waveform (112 655 spikes, Fig. 2a, thick line) contained an upward-going deflection (time lag 0.1 ms) and two downward-going deflections (− 0.2 ms and 0.3 ms), all of which were significantly different from zero (grey-shaded area: 99% confidence interval). The peak-to-peak amplitude of this estimate of the average spike contribution to the local epidural EEG was 79.2 ± 5.0 nV (mean ± s.e.m.). Randomly shuffling spikes, as described above, suppressed these prominent peaks (Fig. 2a, thin line). The peak-to-peak amplitude of this single-spike contribution is far below the (biological and environmental) noise level of about 700 nV observed in on-going signals (signal-to-noise ratio: − 10 dB), explaining why spikes are not directly visible in scalp and epidural EEG recordings.

The triphasic shape of the high-frequency epidural EEG-STA closely resembled the extracellular spike waveforms averaged across all cells (Fig. 2b).

### The amplitude of EEG-STA varies considerably across neurons

The peaks of high-frequency EEG-STA in the grand average could be identified tentatively also at the level of a few single neurons. Despite the large interference from activities of surrounding cells and recording noise, in 6 neurons we found at least one peak that rose significantly above the noise (p < 0.01, two-sample bootstrap test; Fig. 2c). Remarkably, single-cell peak-to-peak EEG-STA amplitudes could be as large as 966 nV — an order of magnitude larger than the amplitude identified in the grand average (79.2 nV in Fig. 2a). However, the shapes of these 6 single-cell EEG-STAs were variable with respect to latency, sign, and peak amplitude (Fig. 2c; mean peak-to-peak amplitude ± standard deviation: 492 ± 323 nV).

**Fig. S1:**
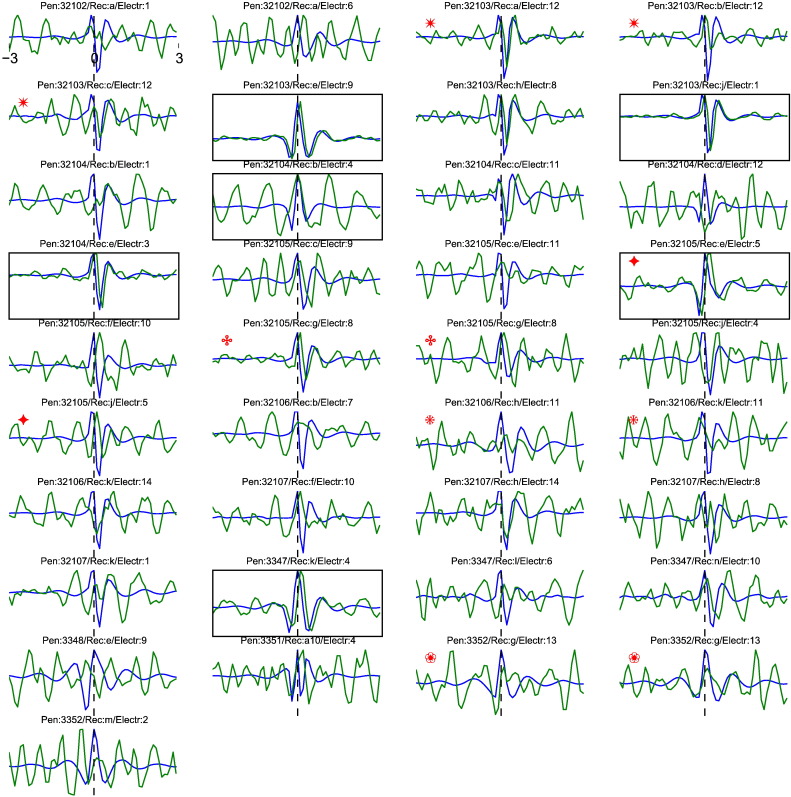
Comparison between spike waveform (blue) and single-cell EEG-STA (green) normalised to unit amplitude. Each panel shows one cell; cells for which EEG-STA peaks were significantly above noise level (cf. Fig. 2b) are highlighted with a black box. Note the striking similarity of waveforms despite a small temporal shift (0.2 ms) in some of the cells. Some EEG-STAs recorded with the same electrode and during the same penetration (grouped by red symbols in the top left corner; penetration, recording and electrode in the title of each panel) differ substantially in amplitude, which disfavours electrical cross-talk.

**Fig. S2:**
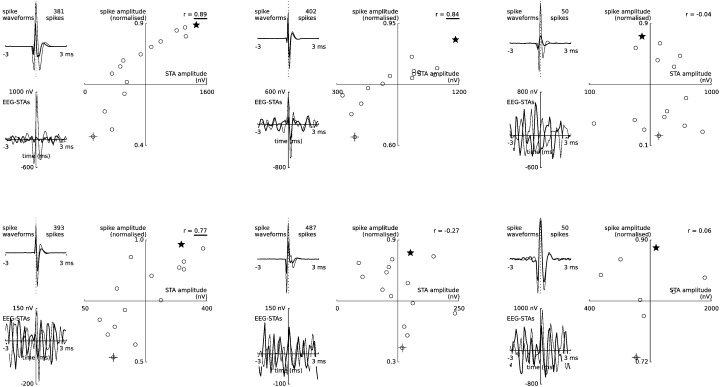
Co-variability between extracellular spike waveform amplitude and EEG-STA for each of the six cells shown in Fig. 2c. Pairs of discriminated extracellular spikes and concomitant spike-triggered EEG segments were split into 15 equally-sized groups according to the peak-to-peak spike waveform amplitude. Scatter plots depict the peak-to-peak amplitude of group-averaged spike waveforms and EEG-STAs; cross and star highlight two samples (light and bold lines, respectively) of spike averages (top left of each panel) and EEG-STA (bottom left). Although all six cells were associated with significant contributions to EEG, EEG-STAs of only three were correlated with variations of spike waveforms (correlation coefficient, *r*, in the upper-right corner; significant *r* underlined, p < 0.05). Positive EEG-STAs/spike amplitude correlations might indicate electrical coupling (‘cross-talk’) between ball- and micro-electrode recording channels.

**Fig. S3:**
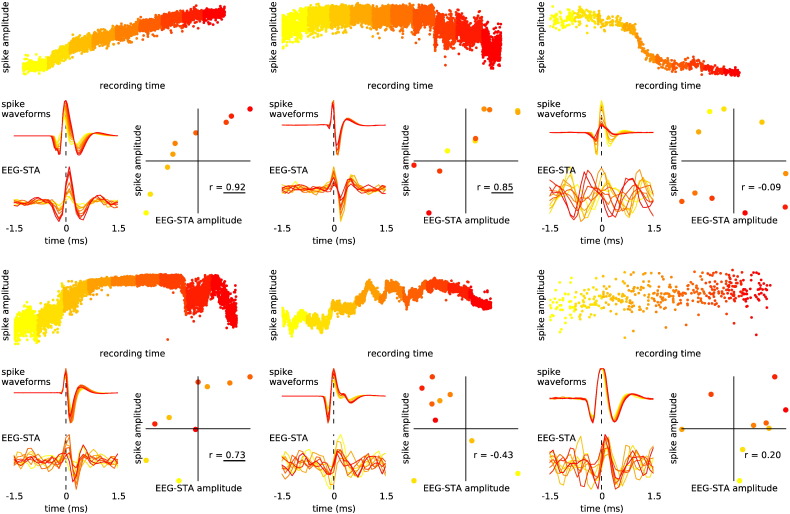
Co-variability of spike and EEG-STA amplitude over time. Each of the 6 panels represents the analysis of a single cell (order as in Fig. 2c). In five of these six cells the spike amplitude varied slowly over time (top) probably reflecting recovery from cell damage or slow drift of the electrode position. We split all spikes of a cell into 10 equally-sized groups according to the time of their occurrence since the beginning of recording session (marked by colours from yellow to red) and averaged both the spike waveforms and the corresponding spike-triggered EEG segments (bottom-left corner). The scatter plots of the peak-to-peak amplitudes of the averages (bottom-right corners; each point reflects a single average; normalised units) indicate a slight correlation (Pearson's correlation coefficient, *r*, given on the graph; underlined values denote p < 0.05). This result suggests that the positive correlations observed in Fig. S2 might be induced by the temporal variations of the spike amplitude.

The shapes of these six EEG-STAs were similar to the shapes of their respective extracellular spike waveforms (Inline Supplementary Fig. S1). This similarity is consistent with spike contribution to EEG, but it might also indicate direct electrical coupling between EEG and spike recording pathways; this is difficult to control for when even nanovolt-level cross talk between amplifiers could have artifactually affected the results reported here. To address this issue, we investigated the spike-to-spike variations of the EEG-STA. If the observed EEG-STA reflects an electrical cross-talk in the recording system, it should follow all spike-waveform variations arising from technical sources, such as noise or the micro-electrode drift. Since the spike waveform amplitude varied slightly over the time of experiment (Inline Supplementary Fig. S3), we could compare the spike waveforms and EEG-STAs averaged over small subsets of spikes. In 3 out of the 6 cells the EEG-STAs and spike amplitudes were positively correlated, so that cross-talk could not be ruled out (Inline Supplementary Fig. S2).

The shapes of these six EEG-STAs were similar to the shapes of their respective extracellular spike waveforms (Inline Supplementary Fig. S1). This similarity is consistent with spike contribution to EEG, but it might also indicate direct electrical coupling between EEG and spike recording pathways; this is difficult to control for when even nanovolt-level cross talk between amplifiers could have artefactually affected the results reported here. To address this issue, we investigated the spike-to-spike variations of the EEG-STA. If the observed EEG-STA reflects an electrical cross-talk in the recording system, it should follow all spike-waveform variations arising from technical sources, such as noise or the micro-electrode drift. Since the spike waveform amplitude varied slightly over the time of experiment (Inline Supplementary Fig. S3), we could compare the spike waveforms and EEG-STAs averaged over small subsets of spikes. In 3 out of the 6 cells the EEG-STAs and spike amplitudes were positively correlated, so that cross-talk could not be ruled out (Inline Supplementary Fig. S2).

Inline Supplementary Figure S1

Inline Supplementary Figure S2

Inline Supplementary Figure S3

Inline Supplementary Figs. S1, S2 and S3 can be found online at http://dx.doi.org/10.1016/j.neuroimage.2014.12.057.

**Fig. S4:**
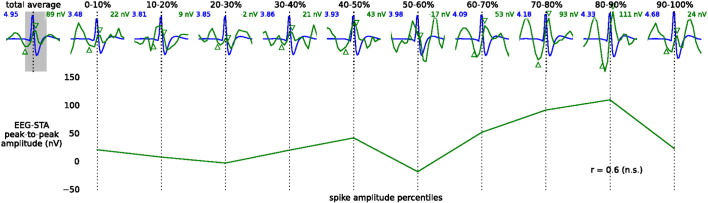
Correlations between spike and EEG-STA amplitude in 34 cells with no significant EEG-STA peak. We sorted single spikes of each cell separately (excluding cells shown in Fig. 2c) from the lowest to highest peak-to-peak amplitude and then split them into 10 groups (amplitude percentiles). We calculated the averaged spike waveform (blue) and EEG-STA (green) within each of the groups and averaged them again across the cells (top insets). The EEG-STA amplitudes in the spike-amplitude percentile groups (value given in top-right corner of each inset) were estimated from the difference of EEG-STA values at latencies aligned with the peak and trough of the overall average across all cells and spikes (total average, top left; a triangle pointing upward/downward marks the trough/peak). The resulting amplitudes are positively, but not significantly (p > 0.05), correlated with the rank of spike amplitude (bottom line; Spearman correlation coefficient, *r*).

Since these cells might have biased the grand-average, we repeated the analysis on a reduced dataset (34 out of 40 cells) where all 6 cells with significant EEG-STA peaks were excluded. The shape of the resulting grand-average EEG-STA of the reduced dataset was similar to that of the full dataset (Fig. 2e); the peaks were smaller but still significant (Table 1, peak-to-peak amplitude: 26.8 ± 6.0 nV, mean ± s.e.m.). In addition, the correlation analysis over subsets of spikes from these 34 cells did not reveal significant correlations between EEG-STA and spike amplitude (Inline Supplementary Fig. S4), thus supporting the interpretation that the reduced EEG-STA represents a depth-to-surface volume-conducted spike contribution.

Since these cells might have biased the grand-average, we repeated the analysis on a reduced dataset (34 out of 40 cells) where all 6 cells with significant EEG-STA peaks were excluded. The shape of the resulting grand-average EEG-STA of the reduced dataset was similar to that of the full dataset (Fig. 2e); the peaks were smaller but still significant (Table 1, peak-to-peak amplitude: 26.8 ± 6.0 nV, mean ± s.e.m.). In addition, the correlation analysis over subsets of spikes from these 34 cells did not reveal significant correlations between EEG-STA and spike amplitude (Inline Supplementary Fig. S4), thus supporting the interpretation that the reduced EEG-STA represents a depth-to-surface volume-conducted spike contribution.

Inline Supplementary Figure S4

Inline Supplementary Fig. S4 can be found online at http://dx.doi.org/10.1016/j.neuroimage.2014.12.057.

As an additional control, we tested the isolation between EEG and micro-electrode recording pathways by calculating a spike-triggered average from an electrode placed in brainstem (pyramidal tract, Pt-STA). This signal shared the data acquisition circuit with the epidural EEG, but it was recorded far from cortical neurons minimizing contributions from volume-conducted spikes. This control Pt-STA did not contain components coincident with spikes, thus corroborating that cortical EEG-STAs were not due to electrical cross-talk within our recording system (Fig. 2d).

### Peripheral stimulation does not affect the EEG-STA amplitude

Our results indicate that the average spike-associated epidural EEG potential is in the order of tens of nanovolts. To obtain this estimate we combined the stimulus-driven and stimulus-free periods of neural activity. Next, we separated these periods and tested whether the EEG-STA amplitude varied across stimulus-evoked and spontaneous conditions.

**Fig. 3:**
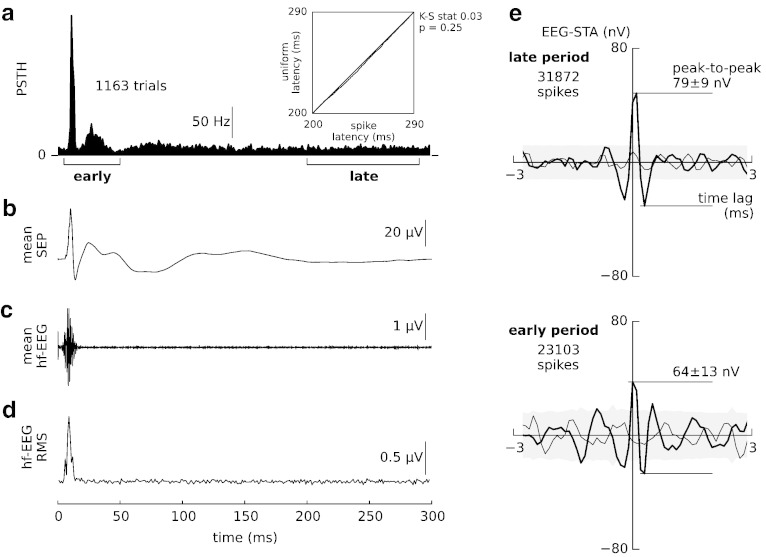
Stimulus-evoked components do not affect the EEG-STA. (a) The post-stimulus time histogram (PSTH, bin width 1 ms; 1163 trials were used in panels a–d) of a single neuron shows a prominent early response (5–50 ms post-stimulus — left bracket). Spike occurrences in the late window (200–290 ms post-stimulus — right bracket) are well approximated by a uniform distribution (inset: quantile–quantile plot comparing the observed spike latencies, abscissa, to uniform distribution, ordinate) confirming the stationarity of spike rate in the late window (Kolmogorov–Smirnov test, p > 0.05). (b) Epidural wide-band somatosensory evoked potentials (SEPs). (c) A stimulus-evoked burst of high-frequency epidural EEG (EEG burst, band-pass filtered, 800–3000 Hz) appears 8–18 ms post-stimulus. (d) Averaging squared high-frequency EEG single trials (RMS — root–mean–square amplitude) to prevent cancellation of oscillations incoherent across trials does not reveal any stimulus-induced activity at post-stimulus latencies > 20 ms. (e) The grand-average EEG-STAs (thick lines) calculated separately for the two post-stimulus periods delimited by brackets in (a): “late” (top) and “early” (bottom). Shuffling spikes across trials removed peaks in both averages (shift predictor, thin lines). No baseline correction was applied to the late-period EEG-STA and its shift predictor.

Neural activity was evoked by median-nerve stimulation, which increased the activity at the single-cell level (post-stimulus time histogram, PSTH, Fig. 3a) and the population level (epidural EEG, Figs. 3b–d). The on-going condition started after the evoked activity returned to baseline: 200 ms after the stimulus spike rate fluctuations already decayed (Kolmogorov–Smirnov test, Fig. 3a with inset), and only negligible fluctuations in the wide-band evoked EEG were present (Fig. 3b), but no evoked (Fig. 3c) nor induced high-frequency EEG (> 800 Hz, Fig. 3d).

The high-frequency EEG-STA based on spikes elicited in the late, stationary window 200–290 ms manifested the very same features as the overall grand-average EEG-STA (Fig. 3e, upper panel). Specifically, its peak-to-peak amplitude (79 ± 9 nV, mean ± s.e.m.) did not deviate from the full EEG-STA (Fig. 2a). After removing evoked EEG components (see the Methods section), we found also little difference in the amplitude of EEG-STA based on early and late spikes (Fig. 3e), which indicates that stimulus has a minor effect on the magnitude of spike contribution to the high-frequency EEG. Therefore, we conclude that the estimated EEG-STA is valid across both, stimulus-evoked and on-going conditions.

### Correlated spikes do not contribute to EEG-STA

If multiple neurons elicited coincident spikes, their contributions might sum and lead to an increase of the observed EEG-STA and an over-estimation of a single-spike contribution to the EEG. We quantified a potential effect of such spikes coincidences on the EEG-STA by analytical calculations.

The neuron density in the macaque cortex area 3b is about 80 000 neurons per square millimetre of cortical surface (O'Kusky and Colonnier, 1982; Collins et al., 2010). Assuming that signals from typical epidural EEG electrodes are dominated by activity generated within a radius of 5 mm, approximately 10^7^ neurons may contribute to the epidural EEG potential. Only a fraction of these cells generates spikes coincident with the reference neuron — the number of such spikes is determined by the cell-to-cell correlations. Experimental measurements of spontaneous correlations between pairs of neurons show that the spike-to-spike synchrony in the cortex is weak and broad (Jermakowicz et al., 2009; Reed et al., 2012; Roy et al., 2007; Baker and Lemon, 2000).

**Fig. 4:**
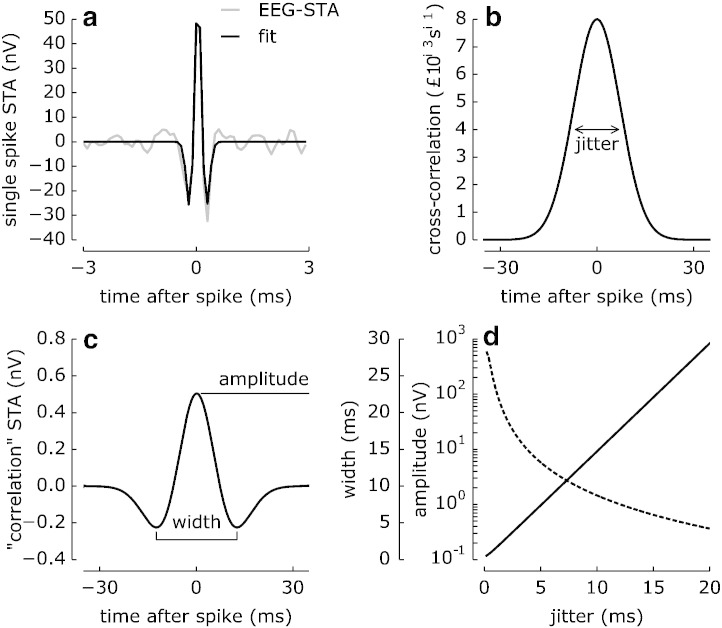
Effects of neuronal correlations on spike contribution to epidural EEG. (a) The grand-average EEG-STA (grey line, same as in Fig. 2a) was fitted with a Mexican hat function (black line, see the Methods section) to approximate the contribution of a *single* spike to epidural EEG (amplitude *A*_STA_ = 60 nV, width *T*_STA_ = 0.45 ms, latency *t*_STA_ = 0.05 ms). (b) Spike cross-correlations are approximated by a Gaussian function with the amplitude *A_xcf_* = 0.008 coincidences/s and width (jitter) *T_xcf_* = 17 ms. (c) The predicted spike-triggered EEG potential from the whole neuronal population (*N* = 10^7^ neurons) that is correlated with the trigger neuron (“correlation” STA) has a shape similar to (a), but it is much broader (width *T*_cSTA_ = 25 ms) and smaller (amplitude *A*_cSTA_ = 0.50 nV). (d) Dependence of “correlation” STA amplitude (dashed line, logarithmic scale) and width (solid line, linear scale) on the width of spike correlations (jitter, cf. b): The STA amplitude rapidly decays with increasing jitter whereas its width increases nearly linearly, corroborating the negligible effect of neuronal correlations on EEG-STA for a realistic jitter magnitude.

To quantify the effect of synchronous spikes on the EEG-STA amplitude, we calculated the expected contribution of all correlated neurons (“correlation” STA) by convolving the estimated EEG-STA (Fig. 4a, black line) with a typical cross-correlogram (Fig. 4b, see Methods). The resulting “correlation” STA (Fig. 4c) is smaller in amplitude by more than two orders of magnitude (0.50 nV) and broader by about factor of 50 (width: 25 ms) than the original EEG-STA (Fig. 4a, grey line). Both, the amplitude and the width critically depend on the temporal scale of correlations (spike jitter, Fig. 4d): Neurons correlated with the trigger neuron could contribute significantly to the EEG-STA amplitude only if the jitter between their spikes was below 3 ms and would match the EEG-STA width only for jitter less than 1 ms — much smaller than indicated by experimental measurements. These analytical calculations support the proposal that the present EEG-STA estimate most likely reflects EEG potentials due to a single spike generated in the recorded neuron.

### EEG-STAs predict the waveform of the evoked high-frequency EEG burst

Estimates of the single-spike contribution to the EEG together with the spiking activity of neurons should allow the EEG activity in response to a stimulus to be predicted. We tested this hypothesis in a paradigm in which neuronal responses were evoked by the electric stimulation of the median nerve (see Methods). Such stimulation triggers a burst of EEG activity that can be recorded epidurally in non-human primates (Baker et al., 2003).

**Fig. 5:**
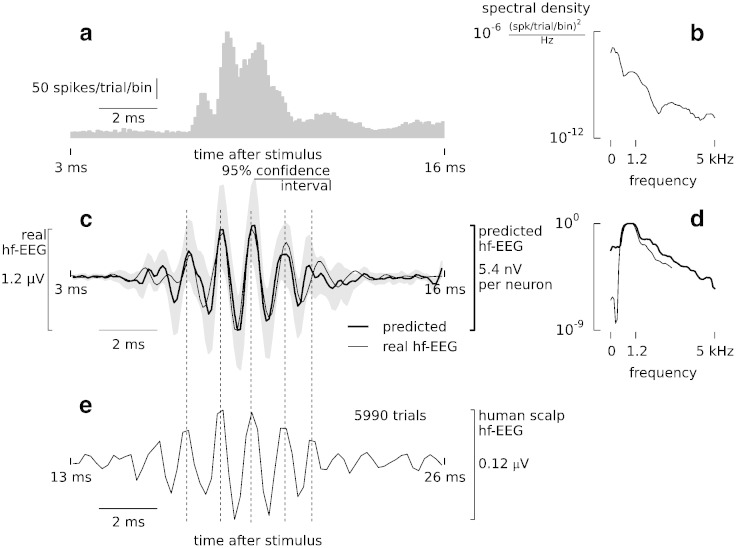
Prediction of high-frequency EEG burst. (a) The post-stimulus time histogram (PSTH, bin width 0.1 ms) averaged across all recorded neurons reveals prominent peaks repeating at high frequency. Spikes were evoked by median-nerve stimulation at *t* = 0 ms. (b) Power spectral density of the population PSTH. (c) The predicted EEG burst due to spike activity shown in (a) was calculated by convolving each single-cell PSTH with its respective EEG-STA (bold line, average across all cells, grey-shaded area — 95% confidence interval). This predicted EEG burst matches the burst recorded simultaneously from the dura (thin line, band-pass filtered 800–3000 Hz, average across sessions). Dashed lines mark the burst peak latencies. (d) Power spectra of predicted and recorded signals (for legend see (c)) match in the high-frequency range (400–3000 Hz). Both spectra were normalised by their maxima. (e) Burst of high-frequency scalp EEG recorded from a human subject in an analogous experimental paradigm (cf. (Waterstraat et al., 2012); stimulation frequency 5 Hz, stimulation intensity 1.5 × motor threshold, 5990 trials, bipolar recording, electrodes C1–C5, band-pass filtered 800–3000 Hz, 10–30 ms post-stimulus). The amplitude of such a burst recorded from the human scalp is one order of magnitude lower compared to macaque epidural recording (bold line in (c)). Note that, owing to conduction delay the response latencies differ, and the burst in (e) is shifted in time accordingly; however, the interpeak latencies (vertical dashed lines) are preserved across species arguing for a similar generator process.

To predict EEG activity, we replaced all stimulus-evoked spikes of a neuron by the EEG-STA of this neuron and averaged them across trials and neurons (Fig. 5). The resulting waveform (Fig. 5c, bold line) matches the actual EEG burst recorded from the dura (Fig. 5c, thin line). The amplitude ratio of the real and predicted EEG burst gives a rough estimate on the number of neurons generating the observed signal. In the present stimulation paradigm, a minimum of about 200 cells are required to generate an EEG burst.

The spectrum of the predicted EEG burst shows the prevalence of high-frequency components (600–1500 Hz) in agreement with the spectrum of the real EEG burst (Fig. 5d, note that the abrupt drop of power at low frequencies is due to the applied high-pass filter at 800 Hz). The spectrum is equal to the product of the spectra of the PSTH (Fig. 5b) and EEG-STA (Fig. 2a, inset).

### EEG-STA amplitude matches theoretical predictions

We finally compared our experimental EEG-STA with previous theoretical analyses of passive propagation (‘volume conduction’) of a spike-related electric field in neural tissue (Murakami et al., 2002, 2003). The dipole moment due to an action potential generated by a layer V pyramidal neuron was found in the range *Q* = 0.78 − 2.97 nA mm (spike-to-baseline amplitude, (Murakami and Okada, 2006)). Using the dipole approximation (Malmivuo and Plonsey, 1995), we calculated the accompanying epidural EEG potential:

(4) $$V_{\mathrm{dipole}}\left(r,\theta \right)=4\frac{\eta }{4\pi }\frac{\mathrm{Qcos}\left(\theta \right)}{r^{2}}\text{,}$$

where *θ* is the angle measured from the dipole axis, *η* is the tissue resistivity and *r* is the distance of the electrode from the source. The neural tissue was assumed homogeneous with mean resistivity *η* = 2.47 Ωm (Logothetis et al., 2007). The factor 4 in front of this equation accounts for the resistivity boundary and the bipolar montage of recording electrodes. The distance from the source was based on the average depth of recorded cells *h* = 2.18 ± 0.83 mm (mean ± std) and the separation between epidural electrodes *d* = 10 mm. Assuming that the electrodes were placed along the axis of maximum potential (*θ* = 0), which for pyramidal neurons nearly coincides with the main neuron axis, the total distance from the source is $r=\sqrt{h^{2}+d^{2}/4}=5.46$ mm and the predicted contribution of an action potential to the local epidural EEG is in the range of *V*_dipole_ = 20 − 78 nV. This theoretical estimate is in good agreement with the experimental results of the present study.

## Discussion

We demonstrated that the onset of a single cortical spike is coincident with an epidural EEG response, in particular, its high-frequency components (800–3000 Hz). A distinct feature of this spike correlate is a sharp (≈ 0.5 ms) triphasic waveform, and the shape resembles extracellular action potentials as recorded intracortically in the vicinity of a neuron. This resemblance indicates that the EEG response might represent a spike that is conducted through the tissue and detected at the dura. The peak-to-peak amplitude of the epidural potential in both stimulus-evoked and on-going conditions is in the order of tens of nanovolts.

This EEG correlate might reflect a single spike but not a group of coincident spikes generated by synchronised neurons. In a theoretical analysis, we showed that the constructive summation of contribution from multiple spikes to EEG > 800 Hz is possible only if their activities are correlated with high temporal precision (< 4 ms). Such high precision is rarely observed among cortical neurons (but see (Denker et al., 2011)) suggesting that the estimated EEG-STA reflects single spikes.

It is impossible to detect the activity of a single neuron or a small group of neurons in on-going EEG, because contributions from typically thousands of neurons are summed. The identification of the correlate of a single spike to the EEG, which is the EEG-STA, was possible due to extensive averaging over a large number of spikes generated during the recording; individual spikes could not be identified in the millivolt-range baseline.

Our experimental setup was optimised for the detection of spike correlates in the EEG; in less favourable conditions (lower sampling frequency, larger electrode size, larger electrode separation, larger distance from neuronal source) spike correlates might be lower in amplitude. Therefore, the reported amplitudes (tens of nanovolts) should be considered as an upper bound on spike correlates in epidural EEG. It is encouraging, however, that this bound agrees well with the theoretical predictions based on simulations of the electric field associated with a spike in a pyramidal neuron and on the biophysics of generation of electric potentials.

### Possible artefacts

Owing to volume conduction, an EEG-STA will be always present, albeit small. The amplitude EEG-STA could be contaminated by technical artefacts such as cross-talk between signal paths of the micro-electrode and epidural EEG recordings. To minimize such interference, the paths were electrically isolated, micro-electrodes were shielded, and separate amplifiers were used for recordings. An additional macro-electrode placed far from the cortical sources (brainstem) to control the effectiveness of the isolation did not show a significant EEG-STA.

A possible indication of a cross-talk might be the co-variation between spike waveform and EEG-STA amplitudes that we found in three neurons. Spike-waveform variations could result from a drift of the micro-electrodes, but in this case they should not affect well-isolated surface recordings.

Alternatively, the co-variation can be explained by cell fatigue, cell damage or accumulation of fluid in the neighbouring tissue; such variations would be picked up by both the micro-electrode and the surface electrode. Specifically, the cell damage is a likely candidate as in these three cells the spike amplitude increased during the recording (Inline Supplementary Fig. S3) possibly reflecting reseal of dendrites damaged during the approach with a micro-electrode.

Alternatively, the co-variation can be explained by cell fatigue, cell damage or accumulation of fluid in the neighbouring tissue; such variations would be picked up by both the micro-electrode and the surface electrode. Specifically, the cell damage is a likely candidate as in these three cells the spike amplitude increased during the recording (Inline Supplementary Fig. S3) possibly reflecting reseal of dendrites damaged during the approach with a micro-electrode.

The positive correlation of amplitudes could also reflect common noise such as environmental noise that contaminates both the micro- and macro-electrode recordings. In the present experimental setup it is not possible to narrow down the source of these correlations to any of these possibilities.

Possible contamination by cross-talk was of lesser concern in the remaining 37 neurons, in which the spike-waveform amplitude was not significantly correlated with the EEG-STA amplitude. In addition, the ensemble EEG-STA averaged across 34 neurons with no significant peaks in their single-cell EEG-STAs contained a peak with an amplitude of 26.8 ± 6.0 nV, which is smaller than reported above but significant and within the same order of magnitude.

### EEG-STA sources

Both amplitude and shape of the spike-associated EEG potential suggest that the principal contribution is generated by action potentials (Murakami and Okada, 2006; Ikeda et al., 2005). In particular, action potentials that back-propagate and invade the dendritic tree may generate strong far-field contributions (Gold et al., 2006). This mechanism is consistent with the polarity of the observed EEG-STA: A positive (negative) amplitude at a pre-central (post-central) electrode suggests that sources are located in deep cortical layers of Brodmann area 3b in the anterior wall of the post-central gyrus and that an EEG-STA might be generated by a spike initiated in the soma and back-propagated towards distal apical dendrites. Correspondingly, this mechanism is assumed to be involved in the generation of somatosensory-evoked 600 Hz oscillations in piglets (Ikeda et al., 2005).

On the other hand, active trans-membrane currents (mainly sodium currents) due to spikes initiated in the soma and propagated forward along the axon could also contribute high-frequency extracellular currents that are picked up by a surface electrode. These contributions of active currents to surface potentials may be negligible because such currents often have a triphasic spatial distribution that produces quadrupolar far-field potentials with a steep fall-off (Clark and Plonsey, 1968). However, the triphasic current distribution of a propagating action potential may be broken by after-hyperpolarizing currents (Storm, 1987), or when the axon bends along its course, or when it runs through a non-homogenous medium (Jewett and Deupree, 1989; Stegeman et al., 1987). Similarly, the symmetry is broken already at the axon hillock where the action potential is initiated; there, the action potential has no trailing repolarization current source. A similar process occurs in reverse at synaptic terminals where the action potential's leading source is truncated just before it terminates (Jewett et al., 1990; Stegeman et al., 1997). Asymmetries caused by any of these phenomena will lead to the generation of currents with a biphasic (‘dipolar’) spatial distribution. Due to a slower fall-off of the amplitude with distance, such dipolar sources are more likely to establish significant far-field components than the quadrupolar potential of a symmetric action potential (Jewett and Deupree, 1989; Stegeman et al., 1987).

### Consequences for broadband EEG

The neuronal firing rate has been shown to be correlated with broadband EEG signals in the frequency range of 30–200 Hz (Crone et al., 2011). Notably, EEG power > 40 Hz contains information about population firing rates in visual cortex encoding a visual scene with millisecond precision (Whittingstall and Logothetis, 2009). Similar results were reported in other brain areas in animal models (Ray and Maunsell, 2011) and even human studies (Manning et al., 2009). The spike contribution to EEG > 800 Hz reported here may shed more light on these results. Spike contributions may produce a broadband power increase similarly to what was already proposed for the synaptic contribution to field potentials (Miller, 2010). In some studies this broadband component of recorded signals is undesirable and spikes are removed by various techniques (Belluscio et al., 2012), but it may be exploited in the EEG recordings for monitoring population firing rates (Miller et al., 2007).

Similarly, a rhythmic spike activity evoked by external stimulation might produce an oscillatory EEG response. At frequencies > 400 Hz the oscillatory response is more likely mediated by spike rather than synaptic contributions (Baker et al., 2003). We demonstrated oscillations in a case study of the 600 Hz EEG response evoked by the median-nerve stimulation (Curio, 2004). Since both the evoked response and the spike contribution were recorded in the same system we could estimate a lower bound for the number of neurons required to generate this ultra-fast macroscopic EEG rhythm.

### Implications for non-invasive electrophysiology

The results presented here have implications regarding the possibility of non-invasive detection of spiking activity using electroencephalography. High-frequency EEG components can be reliably recorded invasively in non-human primates (Baker et al., 2003; Shimazu et al., 2000) and non-invasively in human subjects (Curio et al., 1994; Hashimoto et al., 1996) in response to peripheral stimulation of the median nerve. In the non-human–primate data of the present study, the peak-to-peak amplitude of a typical epidural EEG burst (Fig. 5c, thin line) was in the order of microvolts. In humans, the amplitude of an evoked scalp EEG burst is at least one order of magnitude lower (about 10 times lower in the example shown in Fig. 5e) due to larger distances of the electrodes from the sources and the additional attenuation by scalp and skull. Assuming that the number of responding neurons in the primary somatosensory cortex of humans is approximately 10 times larger than in macaque monkeys (Azevedo et al., 2009), the contribution of a single action potential to the human scalp EEG is predicted to be 100 times smaller than the estimated EEG-STA amplitude, i.e., in the order of 0.2 to 0.7 nV/spike.

Current measurement technology achieves noise levels of 10 $\mathrm{nV}/\sqrt{\mathrm{Hz}}$ (Scheer et al., 2009) or approximately 300 nV when calculated for the width of the EEG-STA frequency band, i.e., 1 kHz. When combined with bounds on scalp EEG-STA amplitude estimated above, the noise levels yield a signal-to-noise ratio of 10log_10_(0.7 nV/300 nV) = − 26 dB for a single spike. However, even such a minute contribution can be detected when a population of neurons is hyper-synchronously activated, as demonstrated for the somatosensory-evoked 600 Hz burst (Curio et al., 1994; Hashimoto et al., 1996).

We argue that also non-synchronous spikes could potentially affect the high-frequency EEG power if a large population is recruited. The resulting variations in scalp or epidural EEG power might be easily monitored using a wideband EEG recording system. Changes in the EEG power originating from summed spike contributions may allow discrimination between excitatory and inhibitory inputs; this is not possible based on low-frequency EEG due to the dependence of EEG polarity on both the type of interaction and the localization of synaptic inputs. Another application for neuroimaging might rely on contrasting high-frequency EEG power between two stimulus conditions to detect small changes in firing rate within local neuronal populations (Jacobs and Kahana, 2010). Further work on the theoretical basis, amplifier and electrode technology, and customized experimental protocols involving spatial and temporal filtering might eventually reduce the single spike detection threshold to a physiological level.

A broader implication of our study is that spiking activity generated by a population of synchronous or asynchronous neurons can contribute significantly to EEG activity at frequencies > 400 Hz. The magnitude of this contribution can be estimated from the amplitude of the reported EEG-STA, the population firing rate, and the expected synchrony.

### Conclusions

Previous studies in somatosensory cortex have identified epidural EEG components > 400 Hz reflecting spike timing of cortical population bursts (Baker et al., 2003) and even of precise temporal patterns detected in cortical neurons (Telenczuk et al., 2011). The results of the present study join the accumulating evidence for spike contribution to field potentials (Manning et al., 2009; Khodagholy et al., 2014; Waldert et al., 2013) and provide a means for quantitative evaluation of these findings in terms of number of contributing neurons and their firing rates. Ultimately, spike-related EEG components may facilitate the interpretation of the high-frequency EEG band in other experimental paradigms and, thus, close the gap between microscopic cellular neurophysiology and macroscopic electrophysiology of the human neocortex.

## Figures and Tables

**Table 1:** Summary table.

Spikes subset	No. cells	No. spikes		Peak-to-peak EEG-STA	
		Total	Per cell median (range)	Grand-average, mean ± s.e.m., nV	Single-cell, mean (range), nV
*Wideband EEG-STA*					
All spikes	40	36 411	652 (76–2915)	–	–

*High-frequency EEG-STA*					
All spikes	40	112 655	1 911 (268–13 993)	79.2 ± 5.0	143 (12–966)
Early spikes (5–50 ms)	40	23 103	393 (28–2506)	64 ± 13	197 (19–946)
Late spikes (200–290 ms)	40	31 872	481 (68–4517)	79 ± 9	166 (22–755)

*Spikes of cells with:*					
– Significant EEG-STA	6	26 162	5812 (416–7318)	267 ± 12	492 (100–966)
– Not significant EEG-STA	34	86 493	1862 (268–13 993)	26.8 ± 6.0	81 (12–256)
